# A Review of Key Biological and Molecular Events Underpinning Transformation of Melanocytes to Primary and Metastatic Melanoma

**DOI:** 10.3390/cancers11122041

**Published:** 2019-12-17

**Authors:** Louise A. Jackett, Richard A. Scolyer

**Affiliations:** 1 Melanoma Institute Australia, 2065 Sydney, Australia; ljackett@gmail.com; 2 Sydney Medical School, The University of Sydney, 2050 Sydney, Australia; 3 Department of Tissue Pathology and Diagnostic Oncology, Royal Prince Alfred Hospital, 2050 Sydney, Australia; 4 Department of Anatomical Pathology, Austin Hospital, 3084 Melbourne, Australia

**Keywords:** diagnosis, melanoma, metastasis, pathology, progression, treatment, melanomagenesis

## Abstract

Melanoma is a major public health concern that is responsible for significant morbidity and mortality, particularly in countries such as New Zealand and Australia where it is the commonest cause of cancer death in young adults. Until recently, there were no effective drug therapies for patients with advanced melanoma however significant advances in our understanding of the biological and molecular basis of melanoma in recent decades have led to the development of revolutionary treatments, including targeted molecular therapy and immunotherapy. This review summarizes our current understanding of the key events in the pathway of melanomagenesis and discusses the role of genomic analysis as a potential tool for improved diagnostic evaluation, prognostication and treatment strategies. Ultimately, it is hoped that a continued deeper understanding of the mechanisms of melanomagenesis will lead to the development of even more effective treatments that continue to provide better outcomes for patients with melanoma.

## 1. Introduction

Melanoma, a malignancy of the pigment-producing cells of the skin, is a major public health problem in most Western countries, where there is a predominance of Caucasians living in temperate climates. Despite awareness that most cutaneous melanomas are caused by ultraviolet (UV) irradiation from sun exposure and public health campaigns to promote “sun smart” behavior, the incidence of the disease has been steadily rising for many decades [1]. In Australia, melanoma is the third most common cancer in men and women and both the commonest cancer and commonest cause of cancer death in young adults [1]. Until recently, there were no effective drug therapies for treating patients with advanced melanoma and most patients with melanoma brain metastases would die within a few months [2]. However, over the past decade, a greater understanding of the molecular basis of melanoma disease pathogenesis and the immune system has led to the development of two new types of drug therapy, molecular targeted therapies and immunotherapies, the latter exerting its effect by harnessing the body’s own immune system to fight the cancer [3]. These therapies have transformed the previously dismal outlook for advanced stage melanoma patients and are now also having a major impact in the management of many other cancer types. Indeed, immunotherapy has been described as cancer’s “penicillin moment”. Melanoma develops from the accumulation of heterogeneous molecular events that have been characterized over recent decades through epidemiological, clinical, pathological and genetic studies [4,5]. The key mutational events, for which ultraviolet irradiation is frequently implicated as a triggering factor, are multifactorial and complex [6,7].

**Figure 1:**
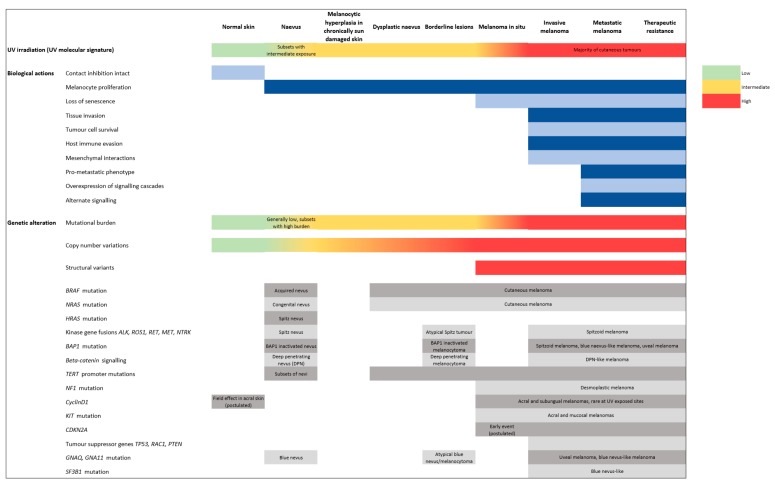
Key phenotypic and molecular events in melanoma pathogenesis and progression. Readers are referred to the text for in depth discussion of each event.

Melanomagenesis was historically conceived to occur in a serial linear model, starting with benign precursors, then moving through ‘intermediate’ lesions, eventually leading to malignant tumors with metastatic potential [4]. However, recent evidence suggests that not only are there multiple pathways by which a melanocyte may transform to a melanoma but also that some of the intermediate steps may be bypassed and that other non-linear biopathways exist [6]. Experimental studies have begun to unravel the aberrant mechanisms that promote melanoma progression and metastasis, including the factors involved in the dysregulation of melanocyte proliferation, impairment of the immune system, and extrinsic agents in the tumor microenvironment that promote primary and metastatic growth (Figure 1) [7]. Accurate biological models of these processes must reflect the great temporal and spatial diversity of the clinical patterns of the disease.

Recently, genetic studies have advanced our understanding of the molecular drivers behind the biological steps of melanomagenesis. Fundamentally, the mitogen-activated protein kinase (MAPK) signaling pathway plays a central role in the development of the vast majority of benign nevi and cutaneous melanomas [8,9]. A single driver mutation of the oncogenes *BRAF* or *NRAS* is a foundation genetic event that activates the MAPK signaling pathway to trigger melanocyte proliferation in approximately 60% of cases [9]. Most of the resulting tumors are benign and remain stable, kept in check by senescence due to functioning tumor suppressor genes [10]. A subset, however, acquire additional molecular alterations such as oncogenic driver mutations and copy number variations that alter tumor suppressor gene regulation [11,12,13]. These events may result in ‘borderline’ or ‘intermediate’ lesions which can mimic melanoma or be precursors of malignant transformation. Ultimately, the hallmarks of fully developed melanoma are the complete loss of tumor suppressor gene function and other mechanisms which confer traits for invasion and metastasis [14,15,16]. In turn, metastatic melanoma may acquire additional mutations that impart treatment resistance to molecularly targeted therapies and immunological agents [17,18,19,20].

This review summarizes our current understanding of the biological processes and molecular events in the pathway of melanomagenesis (Figure 1) and discusses the role of genomic analysis as a potential tool for improved diagnostic evaluation, prognostication and treatment strategies. Ultimately, such an understanding will lead to improved outcomes for melanoma patients.

The authors acknowledge that a comprehensive review of the histopathological diagnosis of melanocytic lesions is beyond the scope of this review and readers are referred to excellent textbooks on this subject [21,22,23].

## 2. Melanocytes in Normal Skin and Early Melanocytic Proliferations

Normal cutaneous melanocytes reside as inconspicuous cells along the basal epidermis, the superficial layer of the skin. Melanocytes possess dendritic processes that provide points of contact with the cell membranes of neighboring keratinocytes, by which the transfer of photoprotective melanin pigment is facilitated [24]. Normal melanocytes maintain uniform cell density relative to other melanocytes and the alteration of this density-dependent regulation is a key developmental event that allows the clustering of proliferating melanocytes in benign nevi and the radial and vertical growth phases of melanoma [25,26].

**Figure 2:**
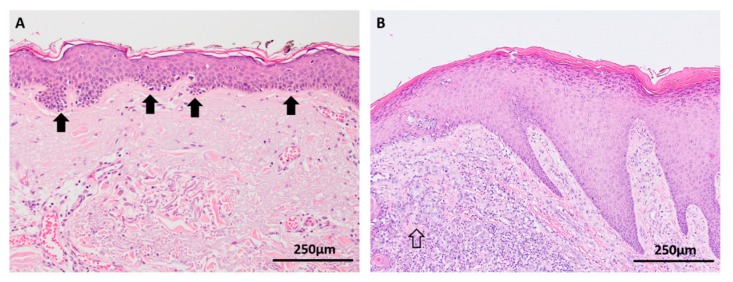
Background skin adjacent to melanomas (haematoxylin and eosin (H&E) images). (**A**) Melanocytic hyperplasia (arrows) in chronically sun damaged skin adjacent to lentigo maligna is a manifestation of a dysregulated single cell microenvironment. Various mutations have been identified in this background skin, many of which are attributed to keratinocytes, but native melanocytes are also postulated to acquire a high mutational burden. (**B**) CyclinD1 amplifications have been detected in melanocytes in epidermis adjacent to acral melanomas (open arrow).

Melanocytic hyperplasia in the epidermis at the edges of lentigo maligna (a type of melanoma in situ occurring on chronically sun damaged skin) is a commonly observed histological phenomenon that is a manifestation of a dysregulated single cell microenvironment and may account for the risk of local recurrence after incomplete wide local excision of melanoma (Figure 2A) [27,28]. However, little is known about the mutational burden of individual melanocytes in sun-damaged skin. Genomic studies have demonstrated an array of various mutations in chronically sun-exposed skin, most of which are likely to be localized to keratinocytes, but it has been postulated that individual native melanocytes may also acquire high mutation burdens [29,30]. In acral skin, multiple gene amplifications (particularly cyclinD1) have been detected among native basal melanocytes in the background skin adjacent to acral melanomas, suggesting that single melanocytes have the ability to accumulate an oncogenic ‘field effect’ independent of being part of a nevus or melanoma in situ (Figure 2B) [31].

## 3. Nevi

**Figure 3:**
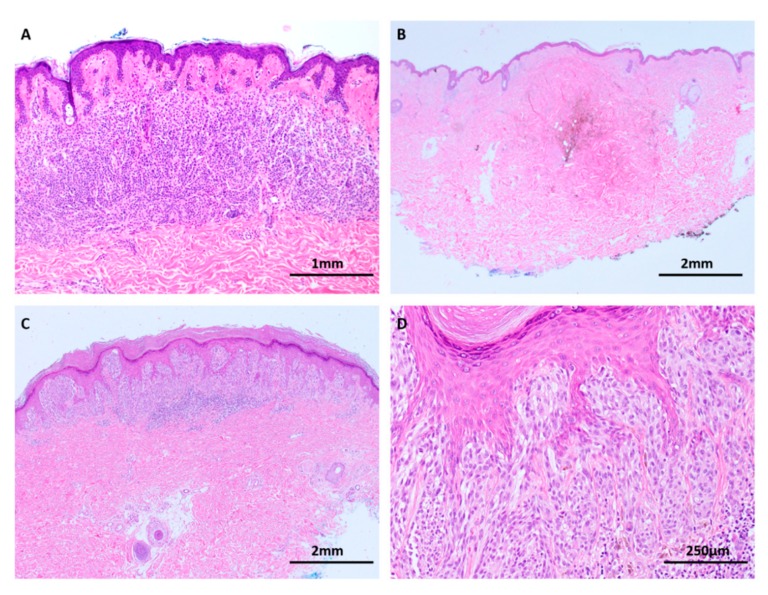
Subtypes of nevi (H&E images). (**A**) The common acquired nevus is a stable lesion resting in a state of senescence and the vast majority possess a low mutational burden. (**B**) Blue nevus. (**C**) Spitz nevus. Both blue nevi and Spitz nevi lack significant chromosomal aberrations by comparative genomic hybridization. (**D**) Some Spitz nevi are associated with isolated gain of chromosome 11p and HRAS mutations, and harbor translocations involving kinase gene fusions.

Nevi are benign clonal proliferations of melanocytes that rest in a state of senescence [32]. They are the most prevalent tumor among humans and are classified into several subtypes based on their clinical and pathological characteristics, the commonest being the common acquired nevus. Other commonly occurring subtypes include the congenital nevus, blue nevus and Spitz nevus (Figure 3). There are observed epidemiological and biological differences among the different subtypes of nevi but contemporary genomic data have shown that nevi are consistently characterized by a lower mutational load than melanomas [16,30,33].

Several studies, including some utilizing whole genome sequencing techniques, have shown that the vast majority of acquired nevi possess single driver mutations of either *BRAF* V600E or *NRAS* Q61R/L without other somatic mutations (Figure 2) [9,34,35,36]. *NRAS* is most frequently observed in congenital melanocytic nevi and is believed to occur in utero [34,37]. In contrast, *BRAF* mutations are more commonly seen in acquired nevi, a characteristic they have in common with many melanomas [34]. Congenital nevi, blue nevi and most Spitz nevi lack chromosomal aberrations, as detected by comparative genomic hybridization [38]. Some Spitz nevi are associated with the isolated gain of chromosome 11p and *HRAS* mutation on that arm, a unique finding that appears to set them apart from melanomas [31]. Many Spitz nevi harbor translocations involving kinase gene fusions involving genes such as *ALK1*, *ROS1*, *RET*, *MET* and *NTRK*.

As noted above, *BRAF* and *NRAS* mutations are also key drivers in many melanomas. However, in contrast to melanomas, which acquire additional driver mutations, nevi enter a suppressive state of replicative senescence which is regulated by the tumor suppressor gene *CDKN2A* via its protein p16, and various transcriptional controls of the cell cycle [33].

Recent molecular studies have unveiled novel findings in benign nevi. Colebatch et al. profiled 14 congenital and acquired nevi by whole exome sequencing and identified subclonal *TERT* promoter mutations, a finding which has not been previously observed in benign nevi [34]. They also demonstrated a subset of nevi with high mutation loads and the UV signatures sbs 7a and 7b, in contrast to the ubiquitous signatures 1 and 5 seen among nevi with low mutation burdens [34]. This finding underscores earlier epidemiological observations that UV irradiation is a common risk factor for both nevi and melanomas, and which hinted at a shared pathway of tumorigenesis caused by the effects of solar UV [39].

The *CDKN2B-CDKN2A* gene cluster is commonly deleted in many types of cancer, and the tumor promoting effects of the *CDKN2A*-encoded p16 and p14 (ARF) tumor suppressors are well studied in melanocytic lesions [11,12,14,40]. The p16 (INK4A) protein binds proteins CDK4 and CDK6, and in this bound state these proteins are unable to stimulate progression of the cell cycle [41]. In contrast, the p14 (ARF) protein reduces the breakdown of p53, a protein that is a key regulator of cell division, senescence and apoptosis [42]. Via these respective mechanisms, p16 and p14 (ARF) ultimately prevent cell division and therefore inhibit tumor progression. The *CDKN2B*-encoded protein p15 is less well studied but has been demonstrated to arrest cell proliferation in melanocytic nevi and its loss promotes melanomagenesis [43]. Changes affecting many other genes are also common, particularly in advanced melanoma, including ARID2, RAC1, and SF3B1, amongst many others [36].

## 4. Borderline Lesions

The concept of ‘borderline’ or ‘intermediate’ lesions was born out of epidemiological and histological observations of tumors with clinically unexpected behavior (such as the frequent lymph node involvement in atypical Spitz tumors despite excellent patient outcomes, including infrequent distant metastasis and mortality), or apparently histologically benign lesions which are associated with an increased risk of progression to melanoma [44,45]. Consequently, the risk profile of such a lesion lies intermediate between overtly benign nevi and unequivocally malignant melanomas. These lesions are unified by their significant diagnostic and management challenges but are biologically heterogeneous. Importantly, their grouping together as ‘borderline’ lesions serves as a theoretical framework for histologic evaluation and further study rather than for nosologic purposes. Examples include dysplastic nevi, Spitzoid neoplasms, atypical blue nevi and related neoplasms, and melanocytomas. Recent genetic investigation has led to significant advances in our understanding of these biologically diverse tumor groups.

**Figure 4:**
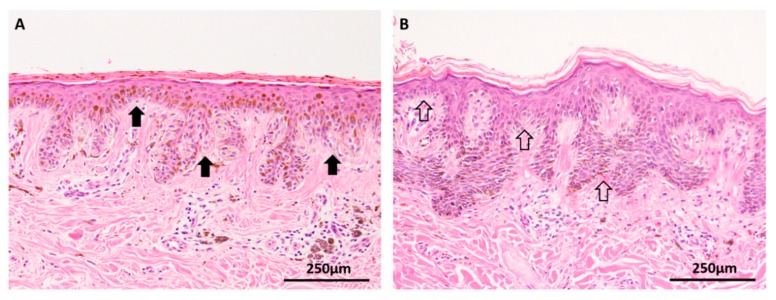
Dysplastic nevi have an overall mutational burden between that of nevi and melanoma (H&E images). (**A**) Multiple dysplastic nevi, such as this example, are clinical markers of increased risk for cutaneous melanoma and diagnosis of lesions with mild to moderate degrees of cytological and architectural atypia (solid arrows) is relatively reproducible. (**B**) Lesions with histological features between severely dysplastic nevus and melanoma in situ, such as this case with focal pagetoid spread and lentiginous architecture (open arrows) among an otherwise nested junctional component, are subject to interobserver and intraobserver variability, even among experts. Identification of differing mutational profiles between dysplastic nevi and melanomas has the potential to assist in diagnosis of these challenging lesions.

The dysplastic nevus has long been recognized as a clinical marker of increased risk for cutaneous melanoma, particularly in patients with more than 10 dysplastic nevi or the dysplastic nevus syndrome [46]. These benign clonal proliferations of melanocytes are clinically, histologically and biologically distinct from other nevus subtypes and have been postulated to represent an intermediate phase between benign nevi and melanoma [16]. Indeed, the diagnostic distinction of severely dysplastic nevus and early melanoma in situ is an enduring problem in histopathology, with well-documented interobserver variability, even among experts (Figure 4) [47,48,49]. Like benign nevi, dysplastic nevi (DN) have been shown to rest in a state of senescence and possess a significantly lower mutational load compared to melanoma [33]. Malignant transformation is occasionally observed in DN but requires the presence of additional alterations in the critical tumor suppressor genes [33]. Recent studies of primary melanomas and their adjacent precursor ‘intermediate’ lesions (dysplastic nevi and melanoma in situ) using a multigene targeted sequencing platform have confirmed that DN lack any alterations in the tumor suppressor genes *CDKN2A, TP53, NF1, RAC1,* and *PTEN*, that they have UV signature patterns distinct from those of melanoma, and that their overall mutation burden appears to be intermediate between benign nevi and melanoma [16,33]. However, the observation that DN tend to harbor non-V600E *BRAF* and *NRAS* mutations suggests that there are likely to be alternative pathways to Clark’s classically postulated model of a linear step-wise progression to melanoma [4,16].

*TERT* promoter mutations, which are present in up to 80% of cutaneous melanomas, have also been observed among dysplastic nevi [16]. This finding and that of Colebatch et al. (above) has put the concept of *TERT* promoter mutations as potential biomarkers for melanoma into question [34]. Nonetheless, the identification of differing mutational landscapes between nevi and melanomas does have the potential to assist with the diagnosis of dysplastic nevi, but validation studies are still required and this approach is currently not appropriate for the routine diagnostic work-up of ambiguous lesions.

**Figure 5:**
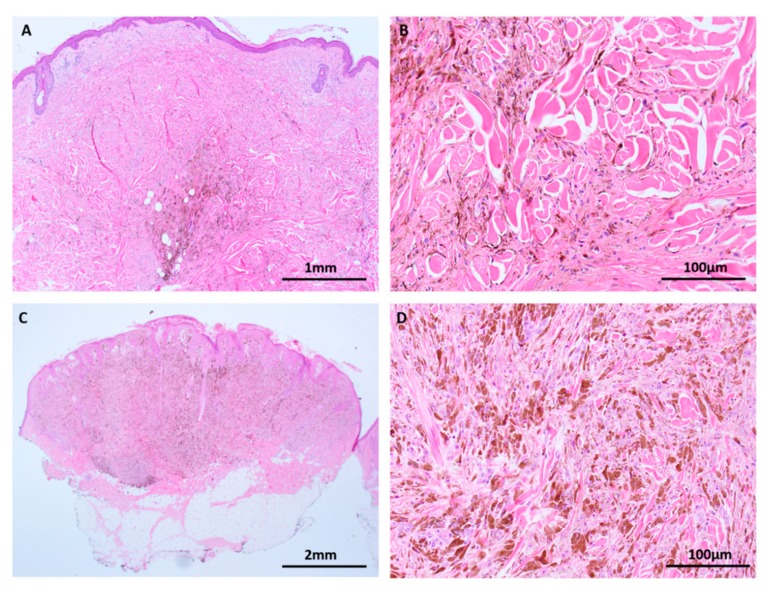
Blue nevi and atypical variants are characterized by *GNAQ* and *GNA11* mutations (H&E images). (**A**,**B**) Blue nevus. The pigmented spindle cells show minimal cytological atypia in benign lesions. (**C**,**D**) This atypical blue nevus shows nuclear atypia, raising suspicion for malignancy but other histological features fall short of melanoma. Recent investigations suggest that *BAP1* mutation is a late event on the pathway to malignancy among blue nevus-like lesions so *BAP1* loss may be a useful ancillary test to support a diagnosis of malignancy in ambiguous cases.

The blue nevus spectrum of lesions includes cutaneous blue nevi, atypical blue nevi and blue nevus-like melanomas. All are characteristically pigment-synthesizing tumors that may be diagnostically challenging (Figure 5). Most blue nevi and related tumors harbor mutations of *GNAQ* and *QNA11*, a characteristic they have in common with uveal melanoma [50,51]. Recently, *BAP1* or *SF3B1* mutations have been found to occur alongside *GNAQ* and *GNA11* in approximately 20% of cases of unequivocally malignant blue nevus-like melanoma, suggesting that this is a late mutational event [52]. *BAP1* loss of expression is a useful ancillary test to supplement histological diagnosis for these lesions.

**Figure 6:**
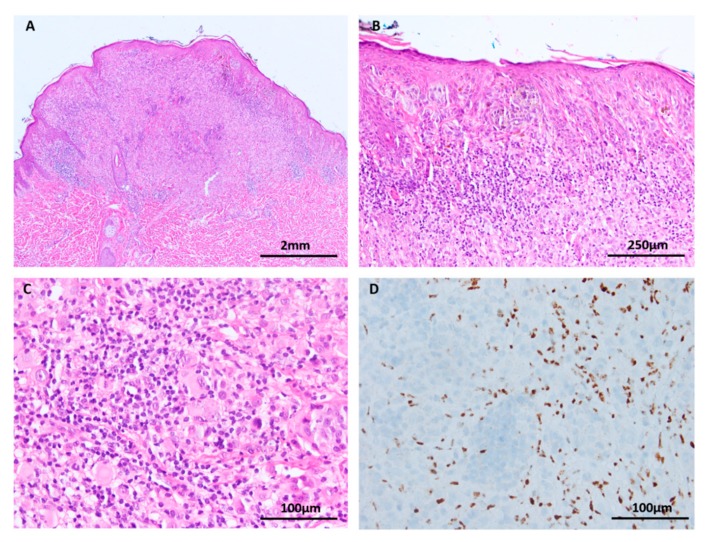
*BAP1* inactivated Spitz tumor (**A**–**C**: H&E images, **D**: *BAP1* immunohistochemistry). (**A**) Low power silhouette of a *BAP1* inactivated Spitz tumor (H&E × 12.5). (**B**,**C**) These lesions often have a characteristic voluminous cytoplasm and a prominent lymphocytic reaction, suggesting a peculiar. (**D**) Lesional melanocytes show loss of nuclear expression of *BAP1*. Lymphocytes serve as a positive internal control.

Atypical Spitzoid tumor, a tumor of uncertain malignant potential that lies between definitely benign Spitz nevus and melanoma, is another lesion that is molecularly complex. A characteristic of all Spitz lesions is the presence of pathways that are distinct from conventional nevi and the majority of cutaneous melanomas [53]. Early in development, Spitzoid tumors harbor *HRAS*, *BRAF* and *BAP1* mutations (Figure 6), as well as kinase gene fusions of *ALK*, *NTRK*, *RET*, *ROS1* and *MET*, and these abnormalities explain the rapid initial growth phase so clinically characteristic of benign Spitz nevi [54]. Atypical Spitz tumors develop aberrations in tumor suppressor genes, including activation of p53 via telomere shortening, and activation of p16 via epigenetic *CDKN2A* regulation. Further accumulation of mutations involving *PTEN*, *ARID2A* and *TERT* appear to promote transformation to a malignant phenotype [55].

Pigmented epithelioid melanocytoma (PEM) is a unique deeply pigmented dermal melanocytic proliferation that overlaps with lesions previously described as epithelioid blue nevus of the Carney complex and many lesions previously termed as animal type melanoma and pigment-synthesizing melanoma [56]. Much like other borderline lesions, these tumors have a clinical behavior and risk stratification intermediate between benign nevi and melanoma. Most of these tumors (80%) show a loss of expression of protein kinase A regulatory subunit 1 alfa (R1alfa), an important cofactor in cyclic adenosine monophosphate (cAMP) signaling involved in melanocyte proliferation, and a mutation that is seen frequently among patients with Carney complex [57].

**Figure 7:**
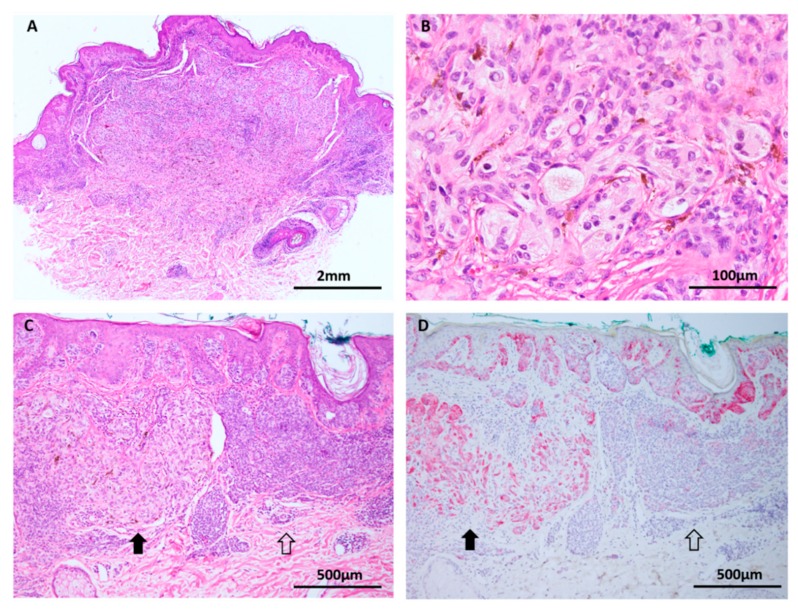
Deep penetrating nevus (**A**–**C**: H&E images, **D**: HMB45 immunohistochemistry). (**A**) Deep penetrating nevi are often seen in conjunction with a conventional nevus (combined nevus). (**B**) Both components harbor MAPK pathway mutations but activated WNT signaling appears to drive transformation to the deep penetrating nevus phenotype with its distinctive large cells, pigment synthesis and lack of maturation. (**C**,**D**) In addition to the distinct genetic differences, the components are delineated by morphology and differing HMB45 expression, with stronger labelling in the DPN component (solid arrows) compared to the conventional nevus component (open arrows).

The deep penetrating nevus (DPN) and its spectrum of atypical variants are another group of heavily pigmented melanocytic lesions that may be confused for melanoma (Figure 7). Atypical DPNs possess some histological characteristics of melanoma but fall short of a diagnosis of overt malignancy. DPNs and atypical DPNs are typified by gain-of-function mutations of beta-catenin and loss of *APC*, both of which exert actions upon the WNT pathway [58,59]. These lesions also possess concurrent MAPK pathway mutations and are often observed to arise in association with conventional nevi (combined nevi), findings which suggest that a beta-catenin mutation is an intermediate step that transforms a conventional nevus into a DPN [60]. This mechanism is likely responsible for the distinctive large cell size, pigmented cytoplasm and lack of maturation of DPN and atypical variants [60]. The vast majority of atypical DPNs are clinically stable; however, malignant behavior is occasionally seen. Indeed, the rare melanomas that share histological similarities to atypical DPNs (termed DPN-like melanoma) also possess the same WNT pathway mutations, suggesting a continuum of transformation [60].

Genetic studies have made significant advances in unravelling many aspects of intermediate lesions and the practical application of ancillary genetic studies has the potential to supplement histological diagnosis, but currently many intermediate or borderline lesions present significant diagnostic and management challenges. Appropriate communication between pathologists and clinicians of estimated risk of aggressive behavior for these ambiguous lesions is of utmost importance. To this end, the term ‘melanocytoma’ has been advocated by the World Health Organization to delineate intermediate lesions with uncertain malignant potential (e.g., atypical DPN and *BAP1* inactivated atypical Spitz tumors) from their clearly benign and overtly malignant counterparts [61].

## 5. Melanoma Arising in Chronically Sun-Damaged Skin

As discussed briefly above, multiple epidemiological and genomic studies have provided evidence for the tumorigenic role of UV irradiation in the development of cutaneous nevi and the vast majority of melanomas [39,62,63]. Early epidemiological observations showed that melanoma has significant regional variation with the highest incidences in New Zealand and Australia, is associated with chronic sun exposure, particularly repeated intense childhood sun damage, and has a higher incidence in individuals who use artificial UV tanning beds [39,62,63]. Genomic studies have subsequently confirmed that melanoma is associated with a high mutational load and mutagenic UV signatures enriched with C > T mutations consistent with the effects of ultraviolet irradiation [13,36,64,65].

The development of melanoma can be divided into the “radial growth phase” (which includes melanoma in situ and the junctional components of invasive melanomas), the “vertical growth phase” and metastasis (local and distant). While this model may erroneously imply a linear progression of disease, it is still useful for conceptualizing the mechanisms of progression.

## 6. Radial Growth Phase Melanoma

**Figure 8:**
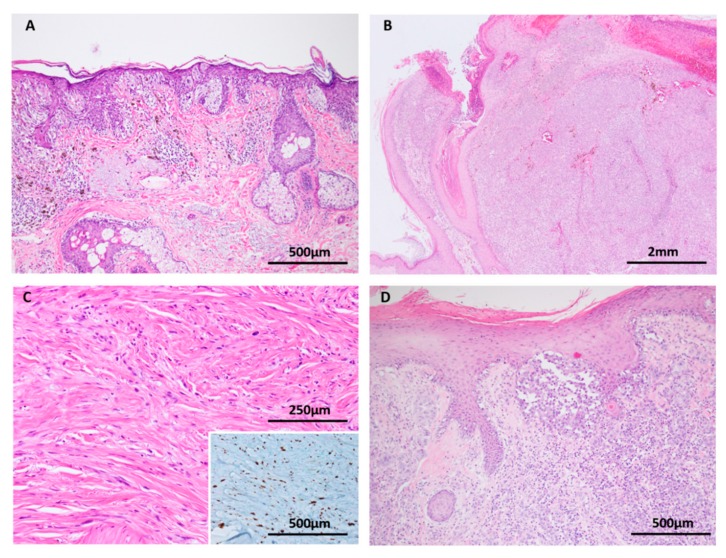
Melanoma (**A**–**D**: H&E images, **C**: inset Sox 10 immunohistochemistry). (**A**) Melanoma in situ is conceptualised as an early melanoma confined by the basement membrane. Loss of contact inhibition is a biological event that is thought to allow the radial growth of melanocytes through the epidermis. *TERT* promoter mutations are identified in melanoma in situ. (**B**) Nodular melanoma. The vertical growth phase of melanoma requires the accumulation of mutations that promote tissue invasion, tumor cell survival, mesenchymal interactions and host immune system evasion. (**C** and inset) Desmoplastic melanomas, shown here with Sox 10 immunohistochemistry, frequently harbor *NF1* mutations. (**D**) Melanomas at sun protected sites, such as this acral melanoma, are biologically distinct from their sun exposed counterparts due to higher frequencies of *KIT* mutations and multiple gene amplifications, commonly cyclinD1.

The loss of contact inhibition has been shown to be an important event in vitro for controlling cell density and is postulated to be critical in vivo to facilitate the radial growth phase of the junctional components of melanocytic lesions, particularly early melanomas (Figure 8A) [25,66,67]. As previously noted, normal melanocytes have dendritic handles that regulate equidistant melanocyte distribution within the basal epidermis. Loss of contact inhibition manifests histologically as lentiginous (“back-to-back”) growth and melanocyte nests. To varying degrees, these features are present in both benign nevi and melanoma in situ, however they are more pronounced in melanoma, manifesting histologically as confluent lentiginous growth, variably sized nests, pagetoid spread and subepidermal clefts [66,67].

Melanoma in situ has been conceptualized as an early melanoma confined by the epidermal basement membrane and, as such, does not have the biological potential to metastasize once it has been completely excised (Figure 8A) [46]. In their study of lentigo maligna cases with and without invasive components, Moreno et al. showed that early lesions had sparse inflammation, while advanced forms of lentigo maligna melanoma had dense lymphocytic infiltrates, suggesting the immunogenicity of melanoma in situ is lower than that of invasive forms of this melanoma subtype [67].

*TERT* promoter mutations have been frequently observed in studies of melanoma in situ but, as noted above, they have also been documented in a subset of nevi with high mutational burdens and dysplastic nevi. *TERT* promoter mutations may therefore be an earlier event in the pathway to melanoma than first thought [16,34]. In contrast, Shain et al. have shown that mutations of *PTEN* and *TP53* are confined to advanced melanomas, but it remains to be elucidated at which point on the spectrum of radial to vertical growth these mutations occur. [16] They have also shown that a minority of intermediate melanocytic lesions, and melanoma in situ, have heterozygous loss of *CDKN2A*, but homozygous loss of *CDKN2A* was only seen in invasive melanomas [16]. Furthermore, it is possible, and indeed likely, that such mutations do not always accumulate in a set order or sequence during malignant transformation.

## 7. Vertical Growth Phase Melanoma

Progression to invasive melanoma requires the accumulation of mutations that continue to allow dysregulated tumor cell proliferation, promote the acquisition of tumor cell survival characteristics and activate advantageous mesenchymal interactions, all while tumor cells evade host immune control (Figure 8B,C) [67,68,69,70,71,72,73,74,75,76].

Mesenchymal interactions at the tumor stroma interface are key determinants of melanoma tumor progression. The malignant stromal phenotype is characterized by protein expression that enhances melanoma survival and progression. One of the earliest observations in melanoma was that of mast cell recruitment to the perilesional stroma by chemotactic factors, such as IL-3, produced by melanoma cells [68]. The subsequent release of growth factors such as the basic fibroblast growth factor (bFGF) and the vascular endothelial growth factor (VEGF) promotes the mitotic activity of tumor cells and tumor neovascularization [68,69]. These mechanisms are underscored by observations that the microvasculature is more concentrated in melanomas than benign nevi, being densest among aggressive melanomas, and that upregulation of VEGF expression, under the influence of the oncogene c-*MYC*, is associated with the progression of melanoma [69,70,71,72,73,74]. Targeting angiogenesis with VEGF-blocking agents has been an effective treatment strategy in several cancer types and its potential role in melanoma continues to be investigated in ongoing clinical trials [75].

Tumor-generated factors have also been shown to play a significant role in the downregulation of protective immune system interactions. In addition to its direct angiogenic effects described above, VEGF has been recognized as an important moderator of immune cell suppression [75]. Other tumor-generated immunosuppressive cytokines include interleukin 10 (IL-10), which reduces the action of melanoma-associated macrophages and lymphocytes [76]. The correlation of high IL-10 expression with melanoma progression provides further evidence that this chemokine is another important influencer in the vertical growth phase of melanoma [76]. Additional and more in-depth immunological interactions are discussed later.

A high expression of the proto-oncogene *c-MYC* has long been identified as a key factor in neoplastic transformation for many tumor types [77]. Well-studied in melanoma, *c-MYC* is implicated in more aggressive vertical growth phase melanomas and progression to metastasis [78,79]. In the appropriate genetic and epigenetic mileu, *MYC* activation leads to aberrations of normal cell proliferation, differentiation, apoptosis and senescence [80,81,82]. These mechanisms result in the hallmark features of tumorigenesis such as unrestrained tumor growth and aberrant protein synthesis, and interactions with the tumor microenvironment to influence vascular growth and dampen the normal response of the host immune system [80]. A necessary feature for sustained and progressive melanoma growth, *MYC* expression can be suppressed by treatment with BRAF/MEK inhibitors leading to tumor shrinkage, manifesting as clinical and/or pathological response; recovery of the overexpression of *MYC* has been shown to drive resistance to these treatments [18].

## 8. Metastatic Melanoma

**Figure 9:**
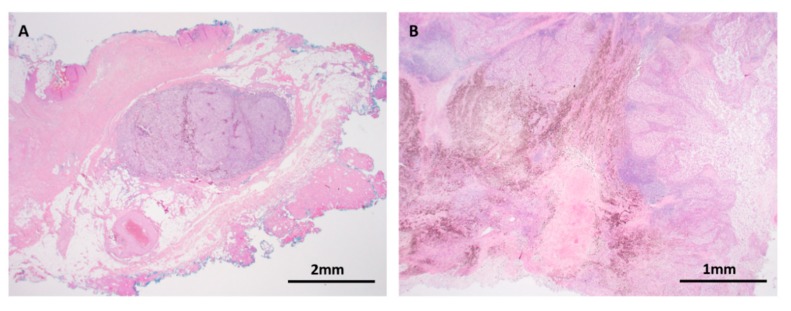
Metastatic melanoma. (**A**) H&E × 12.5. In transit metastasis. Tumor metastasis is dependent on critical factors that drive tumor cell motility, dissemination into angiolymphatics, and tumor cell proliferation away from the primary site. (**B**) H&E × 40. Targeted therapy and immunotherapy have heralded a revolutionary era in melanoma management. Pathological response manifests variably as tumor cell necrosis, melanosis, lymphocytic infiltration and fibrosis, as seen in this lymph node metastasis of melanoma after neoadjuvant therapy. Mechanisms of resistance and primary non-response are the focus of active research.

Tumor metastasis is dependent on critical factors that drive tumor cell motility and dissemination into lymphatics and vascular channels, and that facilitate tumor cell survival and proliferation at distant sites (Figure 9A).

Some of these steps are linear and predictable, such as the reproducible patterns of metastasis to the first lymph nodes in the regional lymphatic basins which were identified in the seminal studies on sentinel node drainage patterns [83]. However, there is evidence that dissemination of melanocytes may also occur via alternative pathways, reflecting the temporally and spatially heterogeneous clinical patterns of metastatic disease [84,85,86,87,88,89,90]. Understanding these cellular processes and the genetic events that underpin them are essential for comprehensive knowledge of the pathogenesis of metastasis and may also provide targets for prognostic biomarkers and therapies.

Tumor-derived exosomes have emerged as important factors in tumor progression [15,84]. Melanoma-derived exosomes influence vascular permeability at potential metastatic sites and alter bone marrow progenitors towards a pro-metastatic phenotype [84]. Exosomes are regulated by the RAB family of proteins, which are highly expressed in melanoma tumor cells [84]. Guo et al. have shown that overexpression of GTPase RAB27A alters the composition of exosomes towards a pro-invasive phenotype, which in turn influences cancer cell movement; this finding correlates with poor survival in a subset of melanomas [15]. Indeed, blockage of exosome contents has been postulated as a potential therapeutic target [85].

Mouse models have shown that the microenvironment of distant sites has an important role in determining whether disseminated tumor cells are eliminated or progress from micrometastases to macrometastases [86]. An example of a chemokine in the microenvironment that influences implantation and growth of metastasis is IL-10, which appears to render lymph nodes susceptible to metastasis through its action on melanoma-associated macrophages and lymphocytes [76].

Peculiar patterns of metastasis sometimes occur that suggest there may be other parallel pathways of progression and inherent differing affinities of tumor cells for particular distant sites. For example, uveal melanomas, which harbor recurrent *BAP1* mutations when they metastasize, have an exceptional tendency to metastasize to the liver [87,88]. This event occurs in up to 90% of patients and may be the only site of metastatic disease [88]. Interestingly, kinetic modelling studies have suggested that melanoma metastasis to the liver may in fact precede the development of a detectable uveal primary by up to 5 years [89]. Clinical knowledge that cutaneous melanomas more commonly spreads to the lung, liver, brain and bone over other sites also hints at potential site specific factors that make certain organs more susceptible to metastasis [90].

## 9. Role of the Immune System

Melanoma is one of the most immunogenic tumors and immunogenicity has been observed to occur prior to the development of invasion [67]. In circumstances of optimal host immunosurveillance, melanoma encounters an early innate immune response that is mediated by interplay of macrophages, granulocytes, dendritic cells and natural killer cells [91,92]. Subsequent to this, the adaptive immune system plays a central role with effector CD4 + and CD8 + T-cells targeting melanoma cells through the actions of interferon-gamma (IFN-γ) or direct cytotoxic interactions [93]. When these mechanisms are appropriately activated, the elimination of the melanoma tumor occurs. However, many melanomas evade these checks and balances and continue to proliferate in an unregulated manner. Considerable effort is currently being dedicated to investigating these processes. Some of the most important mechanisms to have emerged relate to the actions that suppress T-cell function. For example, mutations in MHC class 1 may cause lymphocytes to be ineffective in recognizing melanoma cells [94]. Mutations in CTLA4, PD1 and LAG3 may upregulate immune checkpoints, thereby inhibiting T-cell function [95]. Similarly, the upregulation of ligands PDL1 and PDL2 or the overactivity of regulatory T cells are other key influences of effector T cells [96]. Importantly, these actions can be exploited by novel immunotherapies (discussed later).

The innate immune system also plays a key role in antitumoral immune surveillance and responses. It affects development and growth of melanomas through the release of pro- and anti-inflammatory cytokines and growth factors. Cross-talk between components of the innate and adaptive immune systems, and between immune cells and tumor cells, plays a critical role in tumor maintenance and progression [91,92]. 

There are important differences in the epidemiological profiles of melanomas arising on acral skin (the palms and soles) and at mucosal (internal body) sites, and these early observations provided important evidence in favor of alternative non-UV triggered pathways of melanomagenesis (Figure 1). Genomic studies have subsequently confirmed that acral and mucosal melanomas are biologically distinct from their cutaneous counterparts at sun-exposed sites (Figure 8D). Principally, acral and mucosal melanomas harbor a higher frequency of *KIT* gene mutations and multiple gene amplifications, most frequently of the cyclinD1 gene (Figure 1) [97]. CyclinD1 amplifications, which are infrequent in melanomas arising on sun-exposed sites, occur early in the progression of acral and subungual melanomas [31]. They are also detected among native basal melanocytes in background skin immediately adjacent to acral melanomas, suggesting a ‘field effect’ that may account for an increased risk of recurrent melanomas [31]. Furthermore, acral and mucosal melanomas are characterized by frequent structural variants and lower numbers of point mutations compared with cutaneous melanomas.

## 10. Melanoma Heterogeneity and Implications for Therapeutic Strategies

Targeted therapy against aberrant MAPK pathway signaling has heralded a revolutionary era in melanoma resulting in significantly better anti-tumor activity and survival compared to traditional chemotherapy (Figure 9B). *BRAF-V600* mutation is a robust predictive biomarker for response to selective kinase inhibitors, which can arrest the cell cycle and lead to reduced tumor growth. However, patients almost universally develop resistance to *BRAF*-targeted therapy, usually in the first 12 months of treatment [98]. Approximately 50% of patients treated with dabrafenib or vemurafenib develop disease progression 6 to 7 months after starting treatment [99,100]. Multiple mechanisms of acquired resistance in vivo have been described, including elevated expression of the kinases *CRAF*, *COT1* or mutant *BRAF*, activating mutations in *N-RAS*, *MEK1* or *AKT1*, aberrant splicing of *BRAF* and persistent activation of receptor tyrosine kinases, including PDGFRβ and IGF-1R, and activation of alternate signaling pathways such as Notch1 [20,98,101,102,103,104,105,106,107,108]. Immunotherapy is an alternative treatment modality that harnesses the power of the host’s adaptive immune system by enhancing its own immune surveillance capabilities. To achieve this, anti-PD-1 checkpoint therapies interrupt the PD-1/PD-L1 axis, which in turn releases its inhibitory grip on effector T cells. Disinhibited T cells are therefore able to function against melanoma cells to eliminate the tumor [109]. This therapy has translated into significant clinical outcomes with pathological regression and improved survival occurring in 30–40% of advanced melanoma patients [110,111]. Supplementation with antibody therapy to another immune checkpoint inhibitor, CTLA-4, appears to further enhance clinical outcomes [112]. Responders appear to have activated T-cell signatures with the expression of EOMES, CD69 and CD45RO [113]. Despite promising initial responses, resistance to immune therapies inevitably develops. Early research suggests that an abundance of tumor and host factors are responsible for the varied clinical outcomes achieved by these therapies, such as the interference of CTLA-4, by genetic defects in IFN-γ, and variations in gut microbiota [17,19,114]. Investigation into non-responders and those patients who develop resistance is the interest of considerable active research. Combining immunotherapy with targeted therapy may improve responses but may be associated with unacceptable toxicity [115].

## 11. Biomarker Assays

Knowledge of the molecular makeup of melanoma creates the potential for biomarker assays for a range of settings, such as supplementing histological diagnosis of difficult lesions and improving the early detection of recurrent or metastatic disease.

*TERT* promoter mutations have been shown to correlate with poorer outcomes in subsets of melanoma patients with *BRAF*/*NRAS* mutations, and this may serve as a potential future biomarker. As they are also an early event in the transformation to melanoma, they may also be a valuable diagnostic biomarker for melanoma [116,117].

The evaluation of circulating tumor DNA (ctDNA) assays is a valuable tool for monitoring the response to immunotherapy and appears to be more informative than radiological monitoring in patients with extracranial stage 4 disease [118,119]. Challenges in this area include the considerable heterogeneity among melanoma subtypes, for example the molecular differences between UV associated cutaneous melanomas, non-V600E *BRAF* mutant melanomas, and acral and mucosal melanomas. This implies that ctDNA assays may need to be personalized and targeted to the mutation profile of an individual’s melanoma. Circulating tumor DNA does not appear to be as useful in prognostic modelling in earlier stage disease with current assay sensitivities but may prove to be useful when more sensitive techniques for its detection are employed.

## 12. Conclusions

In summary, melanomagenesis results from the sequential accumulation of key genetic mutations. Alterations of the oncogenes *BRAF* and *NRAS*, which act through the MAP-kinase pathway, are the most frequent early events that drive melanocyte proliferation in both nevi and melanoma. After an initial phase of proliferation, however, nevi rest in a state of senescence, which is influenced by the p16 *Rb* pathway and transcriptional regulation of the cell cycle. Transformation to melanoma requires additional molecular events and characteristically leads to a high mutational burden. *TERT* promoter mutations appear to be an early event, while the inactivation of tumor suppressor genes, such as *PTEN*, *CDKN2A*, *NF1* or *TP53*, drives the later vertical growth phase of advanced melanoma. The key molecular abnormalities that drive progression to metastasis are subjects of ongoing scientific inquiry.

The genomic study of melanocytic lesions continues to prove a powerful tool that has already unveiled a wealth of information and has led to our current comprehensive understanding of melanomagenesis. Continued study of the mutational landscape will continue to unveil potential strategies for therapeutic agents. Furthermore, the observation that nevi and melanoma have low and high mutational burdens, respectively, as well as distinctive UV signatures, has the potential to enhance our diagnosis of histologically ambiguous lesions through the use of sequencing-based technologies.
